# Effects of the methanol fraction of modified Seonghyangjeongki-san water extract on transient ischaemic brain injury in mice

**DOI:** 10.1080/13880209.2021.1941130

**Published:** 2021-06-29

**Authors:** Eun-Jin Kang, Suin Cho, Chiyeon Lim, Byoungho Lee, Young Kyun Kim, Kyoung-Min Kim

**Affiliations:** a College of Korean Medicine, Dong-Eui University, Busan, Republic of Korea; b School of Korean Medicine, Pusan National University, Yangsan, Republic of Korea; c College of Medicine, Dongguk University, Gyeonggi-do, Republic of Korea; d Inju Hospital of Korean Medicine, Incheon, Republic of Korea

**Keywords:** Herbal formula, ischaemic stroke, inflammation, apoptosis

## Abstract

**Context:**

Recently in Korean medicine, the antioxidant and anti-inflammatory activities of Seonghyangjeongki-san (SHJKS) were reported. However, studies on the specific mechanisms of action of SHJKS for the treatment of ischaemic stroke are still lacking.

**Objective:**

This study investigates the mechanism of action of the water extract methanol fraction of modified SHJKS (SHJKSmex) on cerebral ischaemic injury.

**Materials and methods:**

C57BL/6 male mice were orally administered SHJKSmex (30, 100, or 300 mg/kg) for 3 consecutive days (2 days, 1 day, and 1 h, respectively) before middle cerebral artery occlusion (MCAO). Twenty-four hours after MCAO, the infarct volumes were measured, brain edoema indices were calculated, and neurological deficit scores were determined. Inflammation-related substances in the ipsilateral hemisphere were determined by western blotting, dichlorofluorescin diacetate, thiobarbituric acid-reactive substances assay, and enzyme-linked immunosorbent assay.

**Results:**

SHJKSmex pre-treatment at 300 mg/kg decreased infarct volume by 87% and mean brain water content by 90% of the MCAO control group. Moreover, SHJKSmex effectively suppressed the expression of inducible nitric oxide synthase, reactive oxygen species, interleukin 1, and caspases-8 and −9 and increased the B-cell lymphoma 2/Bcl-2-associated X protein ratio (Bcl-2/Bax) in ischaemic mouse brain. The hippocampal pyramidal cell densities were significantly increased in the 300 mg/kg SHJKSmex-administered group compared to the MCAO control group.

**Discussion and conclusions:**

SHJKSmex protected the brain from ischaemic stroke in mice through its antioxidant, anti-inflammatory, and antiapoptotic activities. Our findings suggest that SHJKSmex is a promising therapeutic candidate for the development of a new formulation for ischaemia-induced brain damage.

## Introduction

Stroke is the second leading cause of death worldwide. Its socioeconomic importance is increasing owing to the increasing elderly population and immense socioeconomic burden. As surviving a stroke is accompanied by significant aftereffects, preventing the first stroke is crucial. The primary prevention of stroke involves the management of primary risk factors, such as hypertension, diabetes, atrial fibrillation, smoking, obesity, lack of exercise, and metabolic syndrome, with the exception of uncorrectable risk factors, such as age, sex, race, and family history. However, secondary prevention reduces the risk of stroke recurrence (Johnson et al. 2016).

In this study, a rodent ischaemic stroke model was used to investigate the effects and underlying mechanisms of a modified herbal formula. In rodents, the intraluminal filament transient middle cerebral artery occlusion (MCAO) model, devised by Koizumi et al. (1986) and later modified by Longa et al. (1989), is the most popular of all models. Compared to the direct surgical occlusion of cerebral vessels, the intraluminal filament transient MCAO model is less invasive. Moreover, compared to the embolic model, it is consistent with lesion size and location (Morris et al. 2016; Sutherland et al. 2016). The herbal formula of Seonghyangjeongki-san (SHJKS) is used at the affiliated hospital of the Dong-Eui University College of Korean Medicine in a modified form (SHJKSm), which includes several herbal medicines added to treat stroke (Shin et al. 2016). SHJKS improves cerebral blood circulation, causes the recovery of injured brain cells, resolves blood stasis (Park et al. 2002), reduces the severity of stroke, and improves functional recovery (Choi et al. 2002a).

Many studies on the pharmacological activities of SHJKS have been reported in recent years. Choi et al. (2002b), Kim et al. (2002), Park and Lee (2007), Jeong and Lee (2008), Kim and Kim (2006), and Kim and Lee (2001) determined the neuroprotective effects of SHJKS, and Kim et al. (2006) determined its anti-inflammatory effects. Lee et al. (2000) also investigated its cytoprotective effects against reactive oxygen species (ROS)-induced cell injury.

With respect to cerebrovascular diseases, Kim et al. (2001) determined the protective effects of SHJKS on cellular ion content and metabolism in hypoxic brain cortical cells. Kim and Lee (2001) also revealed that SHJKS decreased infarct volume and edoema in the brain and increased cell survival. Additionally, Lee and Kim (2000) revealed that SHJKS increases cerebral blood flow.

As mentioned above, several studies have investigated the mechanisms by which SHJKS reduces brain damage in ischaemic cerebral infarction. However, the overall mechanisms underlying the reduction in cerebral infarction and cerebral edoema, anti-inflammatory and antioxidant effects, and suppression of apoptosis have not yet been determined in ischaemia-induced brain damage. Additionally, the effectiveness of SHJKSm against ischaemic stroke has not yet been reported. Therefore, we sought to determine the infarct volumes, neurological deficits, brain edoema, aquaporin (AQP)-4 expression, oxidative stress factors, inflammatory mediators, and apoptotic proteins in mice pre-treated with SHJKSm to elucidate the mechanism by which SHJKS inhibits ischaemic brain damage.

## Materials and methods

### Preparation of the aqueous extract of SHJKSm

As SHJKS is used in the modified form (SHJKSm) in Korean medicine, this study also used SHJKSm as the research material. SHJKSm comprises 20 herbs (Table 1); these herbs were purchased between February and May 2018 from a commercial herbal market (Whalim Pharmaceuticals Co., Busan, Korea). All herbs were first checked by Whalim Pharmaceuticals to assess whether each herb met the specifications of the Materia Medica of the Republic of Korea. After purchase, Dr. Cho (School of Korean Medicine, Pusan National University) authenticated its quality in the second order, and the specimens (voucher specimen numbers from 2018-SHJKS-M-01 to 2018-SHJKS-M-20) were deposited and accessioned in Herbarium of Pusan National University School of Korean Medicine to support future researches (specimens from 2018-SHJKS-M-01 to 2018-SHJKS-M-20). All herbs constituting SHJKSm (940.0 g) were prepared in proportions that were 20 times their weight in distilled water and extracted by decoction for 1 h. The solution was then filtered, and the filtrate was concentrated into a powder in a freeze dryer. The final weight of the lyophilised aqueous extract was 202.33 g; thus, the yield was 21.5%.

**Table 1.** Composition of the modified Seonghyangjeongki-san (SHJKSm).

Scientific name (Family name)	Herbal name	Weight (g)	Ratio (%)
*Cyperus rotundus* Linné (Cyperaceae)	Cyperi Rhizoma (香附子)	8	8.5
*Pinellia ternata* Breitenbach (Araceae)	Pinelliae Tuber (半夏)	6	6.4
*Poria cocos* Wolf (Polyporaceae)	Poria Sclerotium (茯苓)	6	6.4
*Morus alba* Linné (Moraceae)	Mori Radicis Cortex (桑白皮)	6	6.4
*Uncaria sinensis* Havil (Rubiaceae)	Uncariae Ramulus et Uncus (釣鉤藤)	6	6.4
*Citrus unshiu* Markovich (Rutaceae)	Citri unshius Pericarpium (陳皮)	6	6.4
*Siegesbeckia pubescens* Makino (Compositae)	Siegesbeckia Herba (莃蘞)	6	6.4
*Agastache rugosa* (Fischer et Meyer) O. Kuntze (Labiatae)	Agastachis Herba (藿香)	4	4.3
*Platycodon grandiflorum* A. De Candolle (Campanulaceae)	Platycodonis Radix (桔梗)	4	4.3
*Arisaema erubescens* Schott (Araceae)	Arisaematis Rhizoma (天南星)	4	4.3
*Areca catechu* Linné (Palmae)	Arecae Pericarpium (大腹皮)	4	4.3
*Aucklandia lappa* Decne. (Compositae)	Aucklandiae Radix (木香)	4	4.3
*Angelica dahurica* Bentham et Hooker f. (Umbelliferae)	Angelicae dahuricae Radix (白芷)	4	4.3
*Atractylodes japonica* Koidzumi (Compositae)	Atractylodis Rhizoma Alba (白朮)	4	4.3
*Perilla frutescens* Britton var. acuta Kudo (Labiatae)	Perillae Folium (紫蘇葉)	4	4.3
*Lindera strichnifolia* Fernandez- Villar (Lauraceae)	Linderae Radix (烏藥)	4	4.3
*Magnolia officinalis* Rehder et Wilson (Magnoliaceae)	Magnoliae Cortex (厚朴)	4	4.3
*Zingiber officinale* Roscoe (Zingiberaceae)	Zingiberis Rhizoma Recens (生薑)	3	3.1
*Glycyrrhiza uralensis* Fischer (Leguminosae)	Glycyrrhizae Radix et Rhizoma (甘草)	2	2.1
*Phyllostachys bambusoides* Sieb. et Zucc. (Gramineae)	Bambusae Sulcus (竹瀝)	5	5.3
Total amount		94	100

The SHJKSm specimens (specimens from 2018-SHJKS-M-01 to 2018-SHJKS-M-20) and the lyophilised aqueous extract of the SHJKSm (Voucher no. 2018Ex-SHJKS-M-01) were deposited at 4 °C and −25 °C, respectively, at the Plant and Extract Bank at Pusan National University School of Korean Medicine for future reference.

### Fractionation of the aqueous extract of SHJKSm in methanol

Since aqueous extracts of herbal medicines contain many solid components that do not have pharmacological activity, it could be difficult to develop formulations that are easily administered orally, especially if the yield is too high. In contrast, alcohol extracts are advantageous in formulations because the yield can be adjusted to obtain low levels. Therefore, in this study, the aqueous extract was further extracted using methanol to obtain a methanol fraction with low yield.

To obtain the methanol fraction of the lyophilised SHJKSm aqueous extract, 100 g of the extract was immersed in methanol (500 mL) and allowed to stand at room temperature for 24 h. Thereafter, the supernatant was retrieved. This process was repeated using the remaining residue. The filtrates obtained after filtering the first and second supernatants through a filter paper were concentrated to dryness, yielding 24.2 g of SHJKSm methanol fraction extract (SHJKSmex); thus, its yield was 24.2%. The total weight of the herbal medicines constituting SHJKSm was 940 g, whereas the obtained methanol fraction of the aqueous extract of SHJKSm was 24 g, with a final yield of 2.6%.

### Drug administration and induction of transient MCAO

Male adult specific-pathogen-free C57BL/6 mice (20–24 g) were obtained from Daehan Biolink Co. (Chungbuk, Korea), and all procedures in this study were approved by the Ethics Committee for Animal Care and Use at Pusan National University (Approval Number, PNU-2017-1752), certified by the Korean Association of Laboratory Animal Care. All efforts were made to minimise animal suffering. The animals were housed in a temperature/humidity-controlled animal facility under a 12 h light/dark cycle and provided food and water *ad libitum* for at least 7 d before the experiment. Animals were randomly divided into five groups: the sham-operated normal group, which comprised mice that underwent surgery but not MCAO (sham control group); the non-SHJKSmex-treated control group (MCAO control group); and the 30, 100, or 300 mg/kg SHJKSmex-pre-treated MCAO groups (30, 100, or 300 mg/kg SHJKSmex-treated groups). Each group comprised at least 23 animals. To administer SHJKSmex to mice, it was dissolved in dimethyl sulfoxide, diluted with 0.9% normal saline, and then filtered through a 0.45 μm pore-size syringe filter to concentrations of 30, 100, and 300 mg/kg.

**Figure 1.**
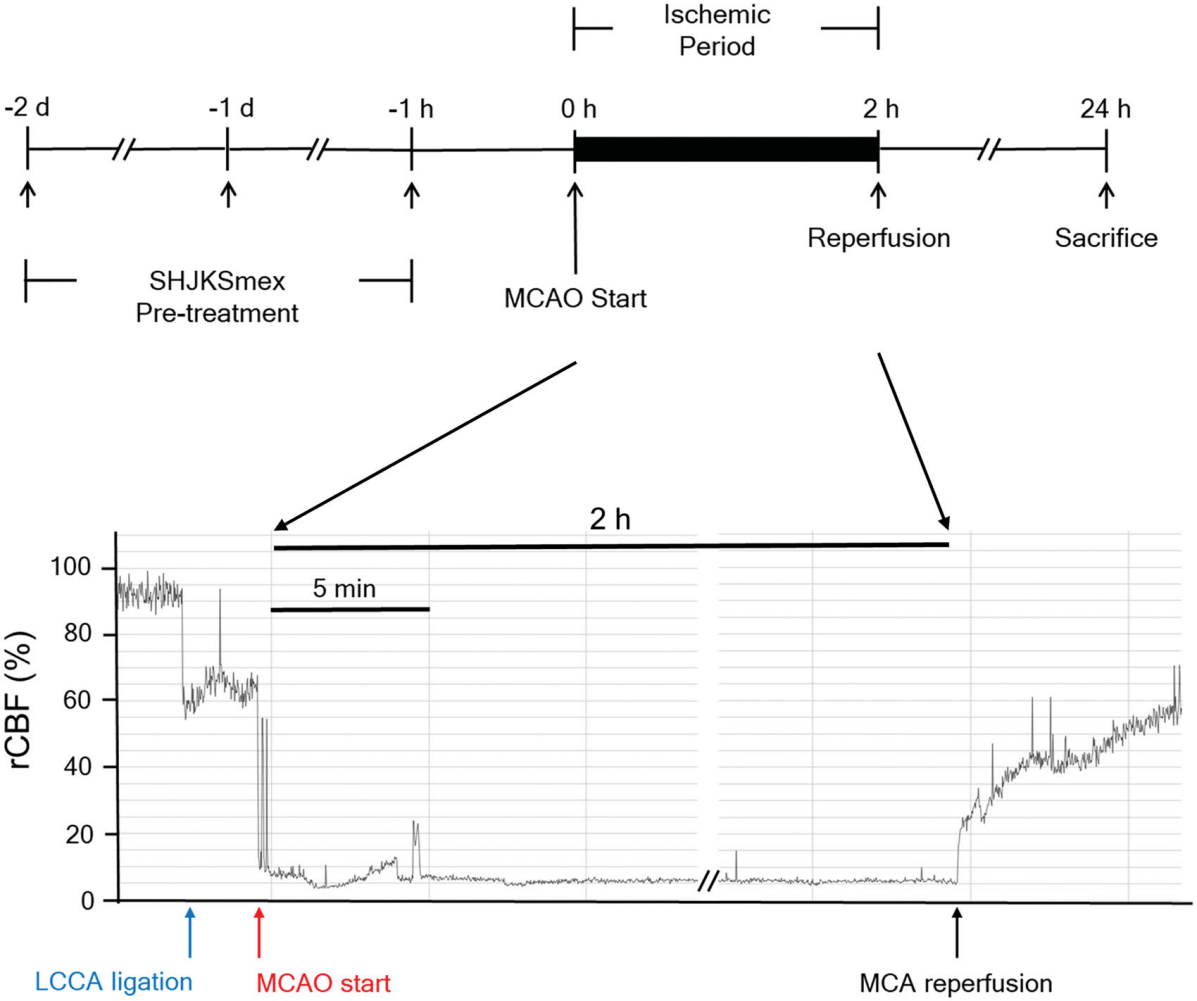
Schematic diagram of the process used to establish the middle cerebral artery occlusion (MCAO) model. The methanol fraction of the modified Seonghyangjeongki-san water extract (SHJKSmex) was administered for 3 consecutive days as pre-treatment before 2 h of MCAO induction. Mice were sacrificed 24 h after the initiation of MCAO (24 h post-MCAO). During the entire ischaemic period, the relative cerebral blood flow (rCBF) was monitored using laser Doppler flowmetry. LCCA: left common carotid artery; MCA: middle cerebral artery.

Before MCAO, the mice in the SHJKSmex-treated groups were orally administered 30, 100, or 300 mg SHJKSmex/kg body weight for 3 consecutive days (2 days, 1 days, and 1 h before the MCAO procedure, Figure 1), and an equivalent amount of normal saline was administered as pre-treatment to the other groups.

Ischaemic stroke was induced by MCAO, as previously described by Jung et al. (2017). Briefly, the mice were initially anaesthetised with 2% isoflurane, and anaesthesia was maintained using 1.5% isoflurane in a mixture of 70% N_2_O and 30% O_2_. The hair on the chest and neck of the animals was removed using a clipper and the skin was cut. After confirming the branches of the left common carotid artery (LCCA), left external carotid artery, and left internal carotid artery under a stereo microscope (Nikon745, Tokyo, Japan), the surrounding connective tissues were carefully arranged to secure a clear view. The LCCA was isolated and an 11 mm long 8–0 mono-filament nylon suture (Ethicon Inc., NJ, USA) was inserted and advanced into the internal carotid artery to occlude the MCA. The inserted filament was fixed to a blood vessel and left for 2 h (ischaemic period). After 2 h, the filament was removed for reperfusion. The relative cerebral blood flow (rCBF) decreased to <20% during the 2 h and increased to >60% of the baseline during reperfusion, indicating the success of the MCAO procedure (Figure 1). The skin was immediately sutured, and the animals were awakened from anaesthesia. A sham operation was performed in the normal group. After the left external carotid artery and LCCA were ligated and the LMCA was not occluded, the incised muscle and skin were sutured. After surgery, the animals were transferred to a cage and maintained at 37 °C for 30 min to allow recovery. During the operation, the rectal temperature was maintained at 36.5 ± 0.5 °C using a heating pad, and rCBF was monitored using a laser Doppler blood flow system (Moor VMS-LDF, Moor Instruments, Devon, UK). The mice were sacrificed using CO_2_ at 24 h or 3 days after MCAO.

### Measurement of rCBF

During the MCAO procedure, rCBF was monitored (Jung et al. 2017) using a laser Doppler flowmeter connected to a single fibre optic probe adhering to the skull surface of the core area supplied by the left. The reduction and maintenance of rCBF under 20% of the pre-ischaemic baseline was used to confirm the occlusion, and rCBF recovery of more than 40% of the pre-ischaemic baseline was considered successful reperfusion.

### Body weight and physiological parameter measurements

The mice were weighed daily during the experimental period, and blood was collected by cardiac puncture under deep anaesthesia 3 d after MCAO. To obtain serum, blood samples were centrifuged at 1,500 × *g* for 15 min at 4 °C. To monitor and exclude potential electrolyte imbalances that might affect the results, the serum concentrations of electrolytes, such as sodium (Na^+^), potassium (K^+^), and chloride (Cl^−^), were measured using an electrolyte analyser (Dri–Chem 3500i, Fuji, Japan).

### Measurement of total infarct volumes and neurological deficit scores

The brains harvested 24 h after MCAO were immediately sliced into 1 mm coronal sections to obtain 10 sections per brain from the olfactory bulb to the area immediately before the cerebellum. Sections were incubated with 2% 2,3,5-triphenyl-tetrazolium chloride (TTC) solution for 17 min at 25 °C and then soaked in 10% neutral buffered formalin for more than 2 h. Total infarct volumes in these sections were calculated using infarct areas as previously described (Jung et al. 2017) with a digital camera (Canon 20 D, Canon Korea, Korea) and ImageJ software (NIH, MD, USA).

At 24, 48, and 72 h after MCAO, neurological deficit scores were determined as previously described (Jung et al. 2017) using the following five-point scale: 0, no neurological deficit; 1, incomplete extension of the right forepaw and reduced grip when the tail was pulled; 2, voluntary movement in all directions and turning to the right when the tail was pulled; 3, walking or circling to the right and sensitive to nociception when stimulated; and 4, no response to stimulation or stroke-related death.

### Brain edoema calculation

Brain edoema indices and whole brain water content were measured to evaluate the effect of SHJKSmex on edoema formation induced by ischaemic stroke. Brain edoema indices were calculated by dividing the injured brain hemispheric areas by the ipsilateral normal brain hemispheric areas, as determined using TTC-stained brain sections. To evaluate edoema formation, the whole brain water content was determined using a previously described vacuum drying method (Sebastiani et al. 2017) and calculated by dividing the brain wet weight by the dry weight.

### Determination of ROS production

Brain ROS production was determined using tissue homogenates with dichlorofluorescin diacetate (Ruan et al. 2013). The tissue homogenate was incubated with 1 mM dichlorofluorescin diacetate for 30 min at 37 °C. The absorbance was measured using a fluorescence microplate reader at an excitation wavelength of 485 nm and an emission wavelength of 535 nm.

To measure ROS stress in the injured brain tissue, the level of malondialdehyde (MDA), a biomarker of lipid peroxidation, was estimated. The level of MDA in the injured hemispheres was examined using the thiobarbituric acid-reactive substances (TBARS) Assay Kit (Cayman Chemical, Ann Arbour, MI, USA) (Dawn-Linsley et al. 2005). The optical density (OD) was measured at 540 nm using a spectrophotometer. The results are expressed as nmol/mg protein content of the wet tissue.

### Inflammatory cytokine analysis

The brain tissue was homogenised in phosphate-buffered saline (PBS; pH 7.4, 5% weight/volume), and the resultant homogenates were centrifuged at 10,000 × *g* for 5 min at 4 °C. Post-mitochondrial supernatants were obtained after an additional centrifugation step at 10,000 × *g* for 20 min at 4 °C and used to perform the enzyme-linked immunosorbent assay (ELISA) (Abcam, Cambridge, MA, USA). The levels of interleukin-1 beta (IL-1β) and tumour necrosis factor-α (TNF-α) in the brain tissue were measured with ELISA using a commercially available kit according to the manufacturer’s instructions. The detection limit of the assay was 0.1 mg/mL. The absorbance of the reaction products was measured at 450 nm using a microplate reader.

### Western blot analysis

The brains of the mice were dissected and homogenised in modified PBS containing 150 mM NaCl, 1 mM ethylenediaminetetraacetic acid, 50 mM Tris, and 1:100 (v/v) proteinase inhibitor. Total protein was isolated using a protein extraction solution (pro-prep, iNtRON, Gyeonggi-do, Korea). Brain tissue lysates were obtained by centrifugation at 13,250 × *g* for 10 min at 4 °C. Proteins were separated on sodium dodecyl sulphate polyacrylamide gels and transferred to polyvinylidene fluoride membranes (Millipore, Darmstadt, Germany), blocked with 5% skim milk in Tris-buffered saline for 1 h at room temperature, and incubated overnight at 4 °C with primary antibodies against AQP-4 (1:1000), inducible nitric oxide synthase (iNOS, 1:500), B-cell lymphoma 2 (Bcl-2) (1:1000), Bcl-2 associated X (BAX) (1:1,000), caspase-8 (1:1,000), caspase-9 (1:1,000), and β-actin (1:500). After overnight incubation, horse radish peroxidase (HRP)-conjugated goat anti-rabbit IgG pAb (1:5000) and HRP-conjugated goat anti-mouse IgG pAb (1:3000) were added for 2 h. Then, the membranes were treated with enhanced luminol-based chemiluminescent substrate solution (GenDEPOT, Houston, TX, USA), and the protein bands were detected on a photosensitive luminescent analyser system (Amersham™ Imager 600, UK). Relative quantities of protein were analysed using the ImageJ program (NIH, MD, USA) against β-actin control.

### Haematoxylin and eosin (H&E) staining

Whole brains were harvested from mice to examine the histological changes induced by ischaemic stroke. The brains were fixed in 10% formalin, dehydrated with alcohol, and embedded in paraffin. The tissue sections were cut into 15 μm thick slices, placed on glass slides, deparaffinized with xylene, and stained with H&E to observe the histological changes in the brain tissue after MCAO injury, under a microscope (ZEISS AXIO, Carl Zeiss, Oberkochen, Germany).

### Statistical analysis

One-way analysis of variance followed by the Holm-Sidak *t*-test *post hoc* analysis was used to determine significant intergroup differences. The analyses were performed using Sigmaplot v12.0 software (Systat Software Inc., San Jose, CA, USA). The results are presented as mean ± standard deviation (SD). Results were considered statistically significant at *p* < 0.05.

## Results

### Effects of MCAO and SHJKSmex pre-treatment on rCBF, body weight changes, and physiological parameters in mice

**Figure 2.**
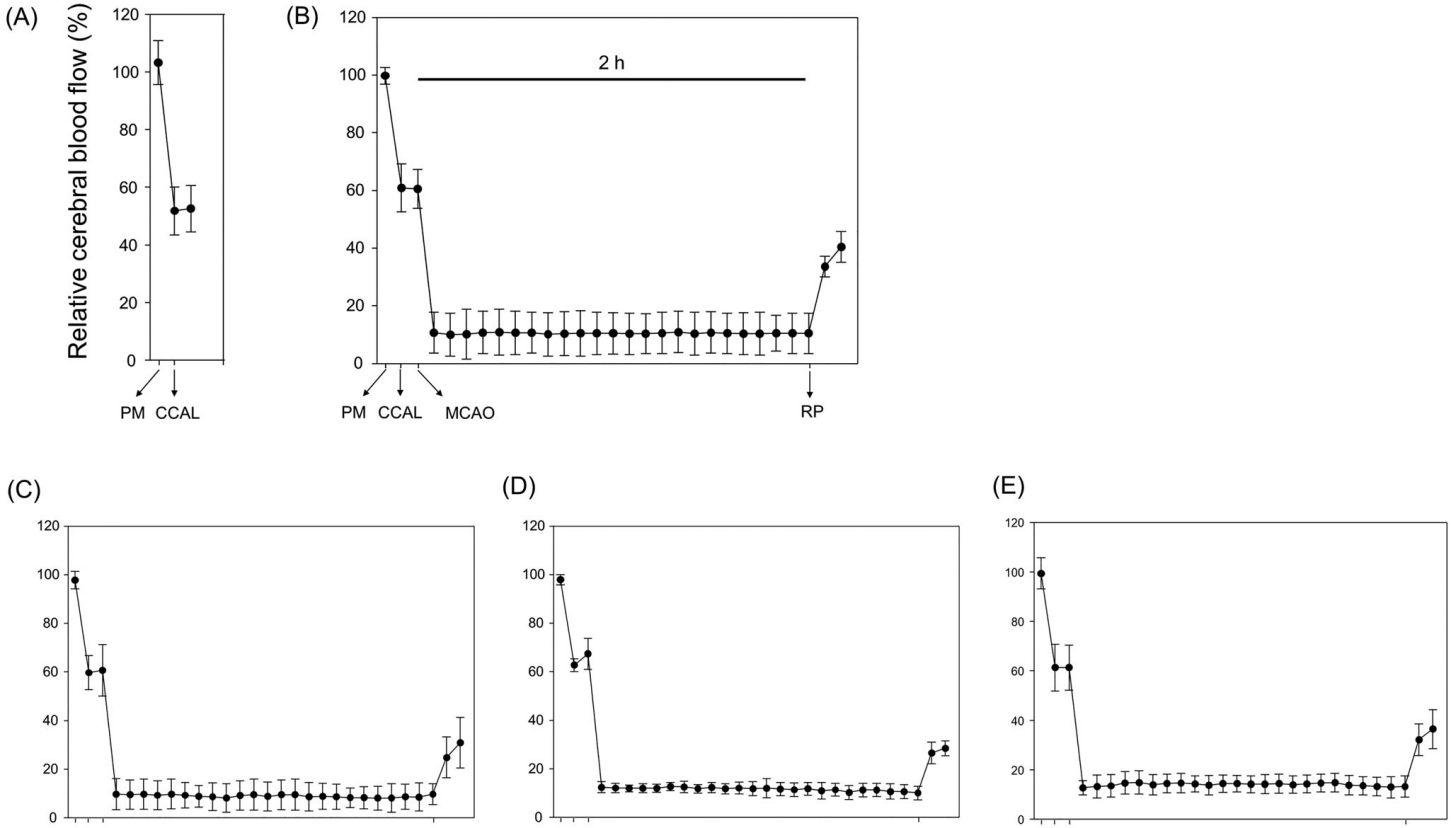
Relative cerebral blood flow (rCBF) in all groups (*n* = 6 per group). (A), Sham-operated normal group; (B), MCAO control group; (C), (D), and (E), SHJKSmex pre-treatment (30, 100, and 300 mg/kg SHJKSmex, respectively) groups. CCAL: common carotid artery; MCAO: middle cerebral artery occlusion; PM: pre-MCAO; RP: reperfusion; SHJKSmex: methanol fraction of the modified Seonghyangjeongki-san water extract.

**Figure 3.**
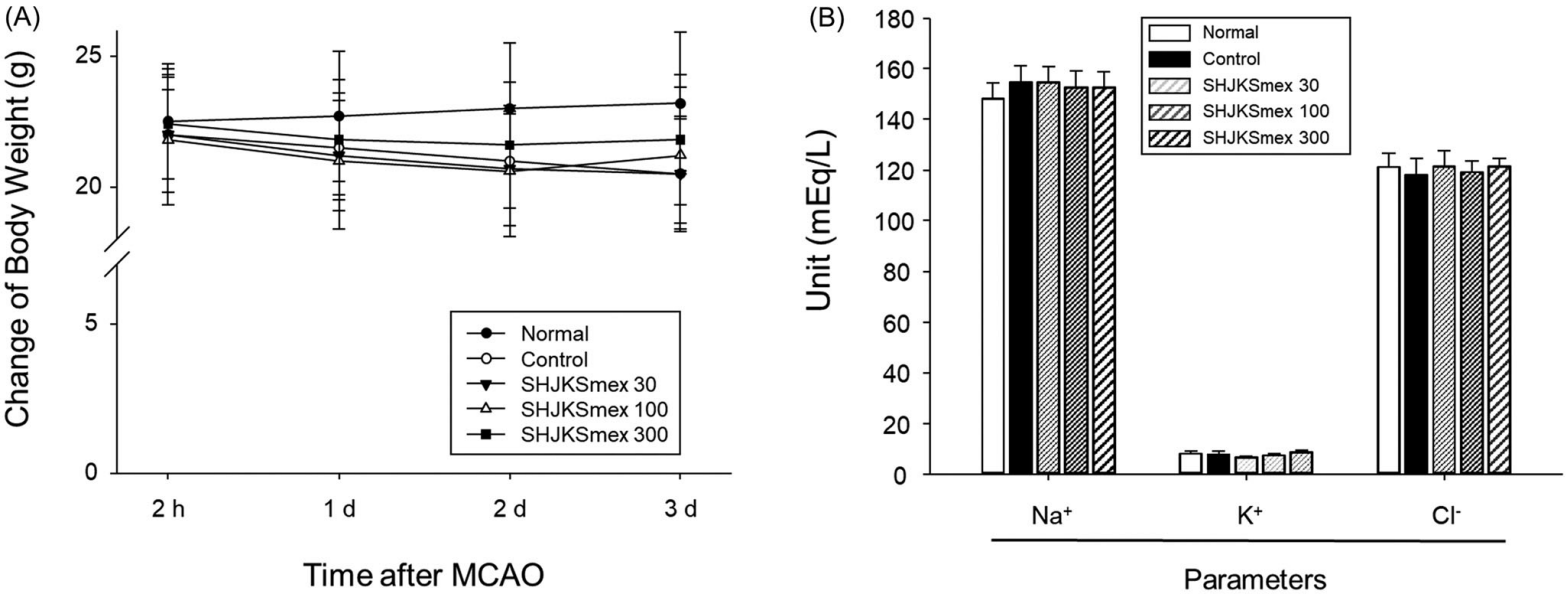
Influence of middle cerebral artery occlusion (MCAO)-induced brain injury and the effect of pre-treatment with the methanol fraction of the modified Seonghyangjeongki-san water extract (SHJKSmex) on body weight changes (A) and physiological parameters (B). Mice were weighed daily during the 4-day experimental period. Serum samples were obtained, and the concentrations of Na^+^, K^+^, and Cl^−^ were measured. Results are presented as the mean ± SD.

There were no significant differences in rCBF among the experimental groups (Figure 2), which means that SHJKSmex pre-treatment did not affect rCBF during MCAO. Moreover, there were no significant differences in the body weight change in the MCAO-operated groups (Figure 3(A)), and no significant changes in the physiological parameters, namely sodium (Na^+^), potassium (K^+^), and chloride (Cl^-^) concentrations, among the groups (Figure 3(B)).

### Effects of SHJKSmex on infarct volumes and neuronal deficit scores

**Figure 4.**
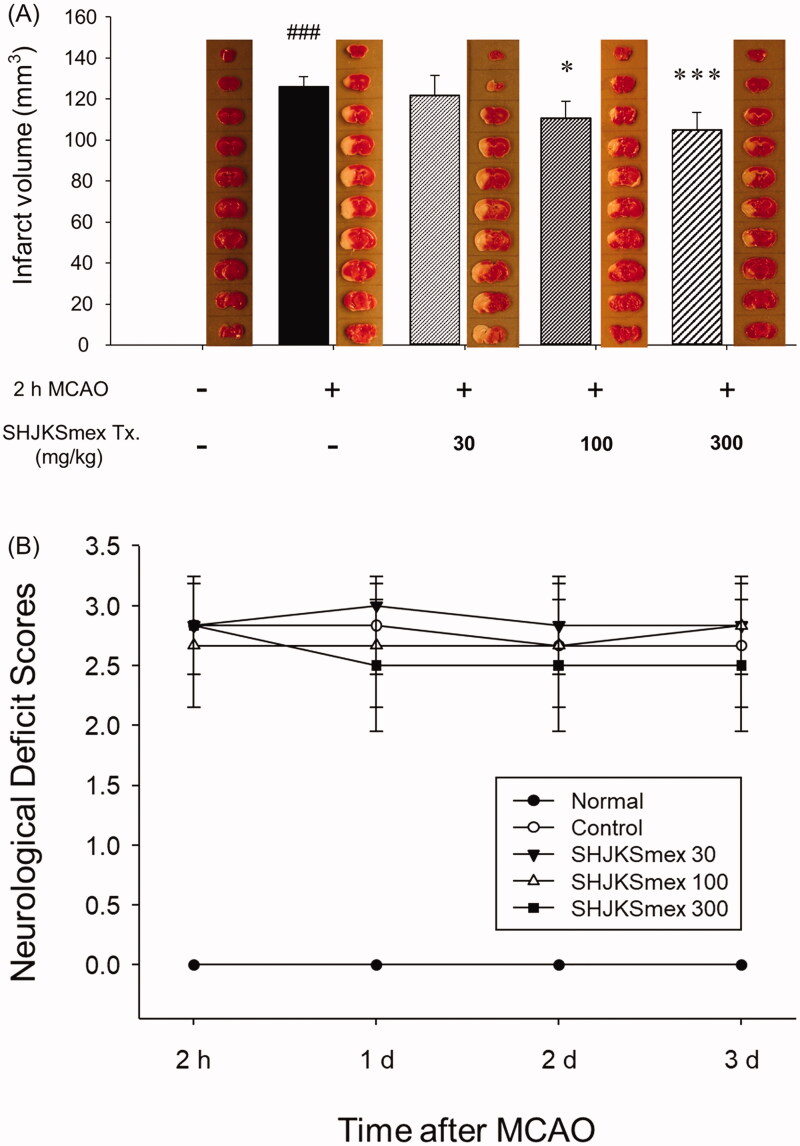
Representative group images and total infarct lesion volumes detected via TTC staining after 2 h of middle cerebral artery occlusion (MCAO) and the effects of pre-treatment with the methanol fraction of the modified Seonghyangjeongki-san water extract (SHJKSmex) on the total infarct volume (A) and neurological deficit scores (B) in each group. The harvested brain slices were stained with TTC to measure the infarct volumes. Ischaemic regions were identified as pale regions in the coronal slices. SHJKSmex pre-treatment significantly decreased the infarct volumes at 24 h after MCAO. SHJKSmex pre-treatment did not improve the neuronal deficit scores at 24 h after MCAO. The results are presented as the mean ± SD. ^###^*p* < 0.001 vs. normal group, *p < 0.05, ***p < 0.001 vs. MCAO control group; *n* = 6 in each group. TTC: 2,3,5-triphenyl-tetrazolium chloride.

The infarct regions were determined using TTC staining. The sham operation caused no damage; however, MCAO caused considerable (*p* < 0.001) damage in the ipsilateral hemispheres (126 ± 4.98 mm^3^). Significantly smaller infarct lesions were found in the 100 or 300 mg/kg SHJKSmex-pre-treated MCAO groups (*p* < 0.05, and *p* < 0.001, respectively) than in the MCAO control group (100 mg/kg, 110.8333 ± 8.1343 mm^3^; 300 mg/kg, 105 ± 8.579 mm^3^; Figure 4(A)). Furthermore, neuronal deficit scores were significantly (*p* < 0.001) higher in mice subjected to MCAO. Pre-treatment with SHJKSmex caused no significant reduction in motor behavioural scores relative to the MCAO controls (Figure 4(B)). The total cerebral infarction volume and neurological deficit scores were shown to be irrelevant through short-term (24 h after MCAO induction) observations; therefore, in our subsequent studies, we plan to monitor long-term behaviour to determine whether the neurological deficit scores are improved by SHJKSmex administration.

### Effects of SHJKSmex on brain edoema and AQP-4 expression in ipsilateral brain tissue

**Figure 5.**
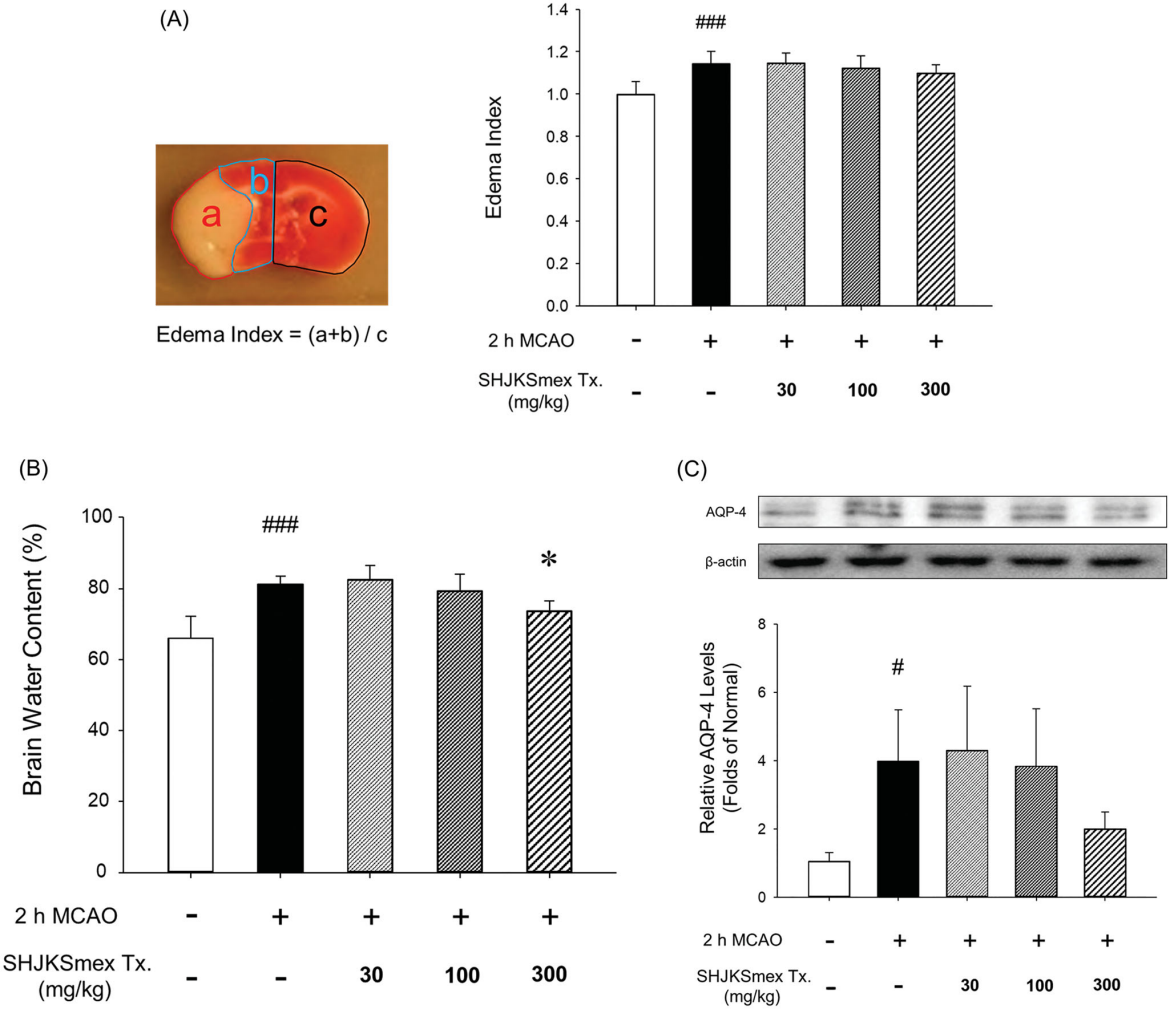
Influence of middle cerebral artery occlusion (MCAO)-induced brain injury and the effects of pre-treatment with the methanol fraction of the modified Seonghyangjeongki-san water extract (SHJKSmex) on brain edoema indices (A), brain water content (B), and aquaporin-4 (AQP-4) protein expression (C) in the brains of mice with MCAO. Pre-treatment with SHJKSmex caused no significant changes in the brain edoema index. Pre-treatment with 300 mg/kg SHJKSmex significantly suppressed the whole brain water content. SHJKSmex pre-treatment had no suppressive potential on AQP-4 overexpression; however, the values were considered to display a decreasing tendency. Western blots and quantitative analysis of AQP-4 protein expression in the brain tissue revealed similar results to the brain edoema index and brain water content findings. Results are presented as the mean ± SD. ^#^*p* < 0.05, ^###^*p* < 0.001 vs. normal group, *p < 0.05 vs. control group; *n* = 6 per group.

Brain edoema indices were calculated by dividing the brain into three parts (Figure 5(A)). The mean brain edoema index remained unchanged in the SHJKSmex group (Figure 5(A)). However, the mean brain water content was significantly (*p* < 0.05) lower in the SHJKSmex 300 mg group than in the control group (Figure 5(B)). Both the brain edoema index and water content can be used to measure brain edoema induced by brain injury. In this study, measuring the amount of water contained in the brain was found to be slightly more specific for edoema than measuring the brain edoema index using TTC staining.

SHJKSmex pre-treatment was found to have no significant suppressive effect on AQP-4 overexpression; however, the values demonstrated a decreasing tendency (Figure 5(C)).

### Effects of SHJKSmex on ROS production and lipid peroxidation

**Figure 6.**
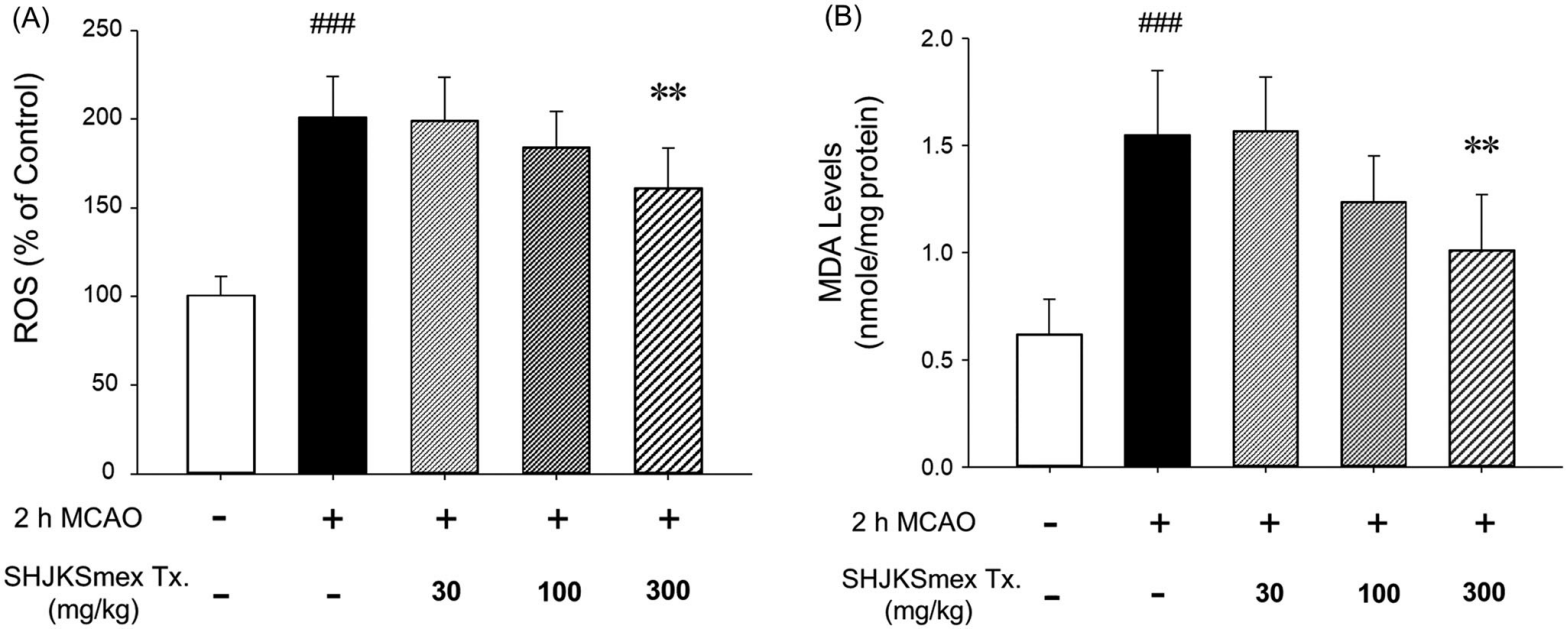
Effects of pre-treatment with the methanol fraction of the modified Seonghyangjeongki-san water extract (SHJKSmex) on reactive oxygen species (ROS) (A) and malondialdehyde (MDA) (B) levels in the brains of mice with middle cerebral artery occlusion (MCAO). Pre-treatment with 300 mg/kg SHJKSmex significantly lowered ROS and MDA levels. Results are presented as the mean ± SD. ^###^*p* < 0.001 vs. normal group, **p < 0.01 vs. control group; *n* = 6 in each group.

To examine the antioxidant effects of SHJKSmex, we measured the levels of ROS and MDA in the brain. The ROS and MDA levels were significantly (*p* < 0.001) elevated in the MCAO-induced group (ROS, 195.40 ± 20.37%; MDA, 1.53 ± 0.33 nmol/mg protein) compared with the sham-operated group (ROS, 100.80 ± 11.65%; MDA, 0.60 ± 0.18 nmol/mg protein). However, significantly lower ROS and MDA levels (*p* < 0.01) were observed in the 300 mg/kg SHJKSmex group than in the MCAO control group (ROS, 156.40 ± 21.81%; MDA, 1.06 ± 0.26 nmol/mg) (Figure 6). Therefore, SHJKSmex was confirmed to reduce the levels of MDA, a product of fatty acid peroxidation, by inhibiting ROS production and is assumed to minimise brain damage through these mechanisms.

### Effects of SHJKSmex on iNOS expression and pro-inflammatory cytokine levels

**Figure 7.**
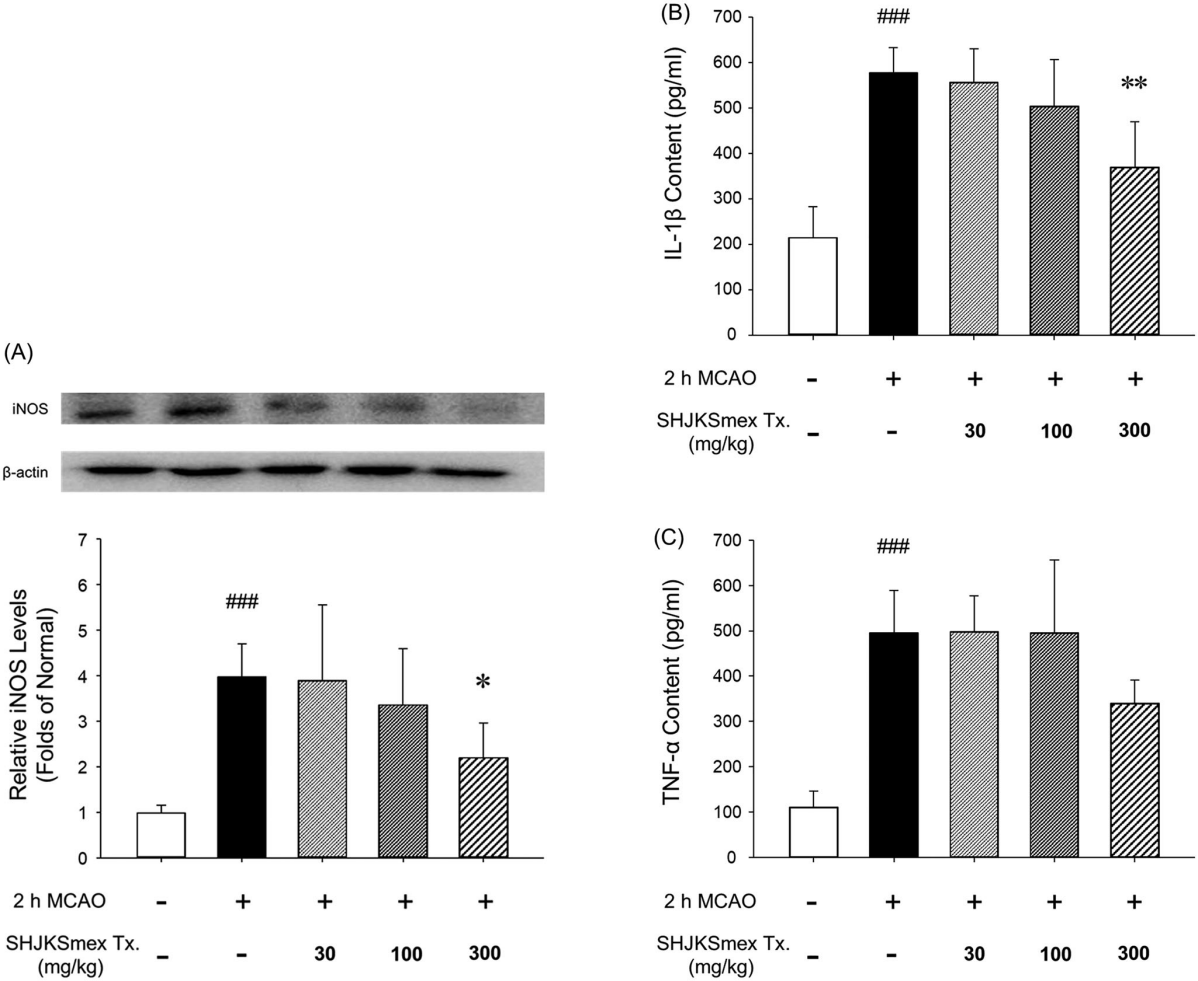
Effects of pre-treatment with the methanol fraction of the modified Seonghyangjeongki-san water extract (SHJKSmex) on inducible nitric oxide synthase (iNOS) (A), interleukin (IL)-1β (B), and tumour necrosis factor (TNF)-α levels (C) in the brains of mice with middle cerebral artery occlusion (MCAO). SHJKSmex pre-treatment significantly decreased iNOS levels in the mouse model of ischaemic brain stroke. Representative western blots and quantitative analysis of iNOS expression demonstrate the effect of SHJKSmex on iNOS expression in the brain tissue. IL-1β and TNF-α levels were measured using a commercially available ELISA kit. Results are presented as the mean ± SD. ^###^*p* < 0.001 vs. normal group, *p < 0.05, **p < 0.01 vs. control group; *n* = 6 in each group.

Western blot analysis was performed to identify protein changes in the harvested brain sections 24 h after MCAO. Based on our findings, inducible NOS (iNOS) levels were significantly (*p* < 0.001) higher than those in the sham controls. However, the mice in the 300 mg/kg group had significantly (*p* < 0.05) lower iNOS levels than those in the MCAO control group (Figure 7(A)).

The effects of SHJKSmex on brain inflammation after MCAO-mediated injury were evaluated by measuring the levels of pro-inflammatory cytokines. The brains of mice with MCAO exhibited higher (*p* < 0.001) concentrations of IL-1β (577.60 ± 55.97 pg/mL) and TNF-α (495.80 ± 93.45 pg/mL) than the sham-operated mice (IL-1β, 214.80 ± 68.65 pg/mL; TNF-α, 111.20 ± 34.75 pg/mL). However, SHJKSmex pre-treatment significantly (*p* < 0.01) suppressed the expression of IL-1β (300 mg/kg, 369.80 ± 100.75 pg/mL; Figure 7(B)). Although not significant, the expression of TNF-α was markedly decreased in the 300 mg/kg group (300 mg/kg, 340.20 ± 50.72 pg/mL; Figure 7(C)). The above results confirmed that SHJKSmex reduced brain damage by suppressing inflammatory reactions.

### Effects of SHJKSmex on apoptosis-related protein expression in the brain tissue

**Figure 8.**
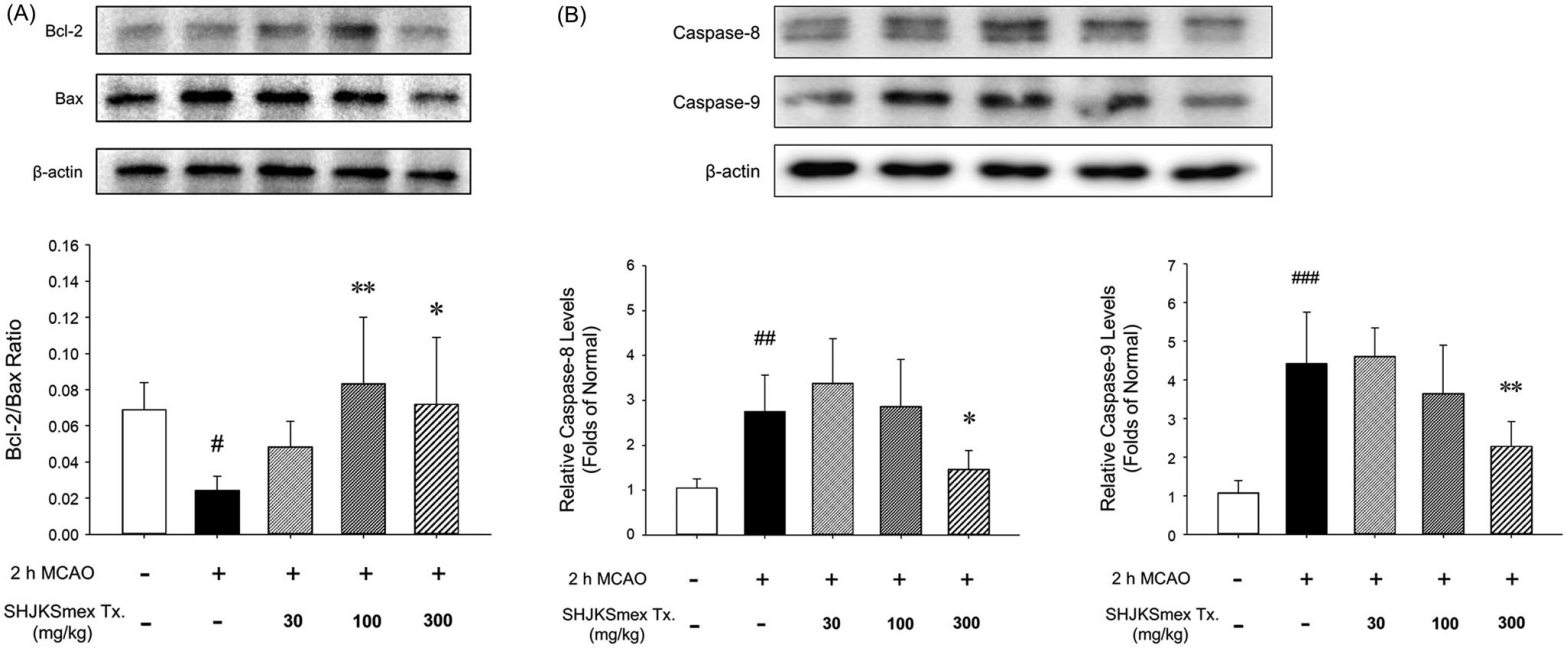
Effects of pre-treatment with the methanol fraction of the modified Seonghyangjeongki-san water extract (SHJKSmex) on the Bcl-2/Bax ratio (A) and caspase-8 (B) and caspase-9 (C) protein expression in the brains of mice with middle cerebral artery occlusion (MCAO). SHJKSmex pre-treatment significantly increased the Bcl-2/Bax ratio in the mouse model of ischaemic brain stroke. Representative western blots and quantitative analysis of Bcl-2 and Bax protein expression (A) revealed the upregulating potential of SHJKSmex on the Bcl-2/Bax ratio in the brain tissue. Representative western blots and quantitative analysis of caspase-8 and caspase-9 protein expression (B) revealed the potential of SHJKSmex to downregulate caspase-8 and caspase-9 protein expression in the brain tissue. Results are presented as the mean ± SD. ^#^*p* < 0.05, ^##^*p* < 0.01, ^###^*p* < 0.001 vs. normal group; *p < 0.05, **p < 0.01 vs. control group; *n* = 6 in each group.

Western blot analysis showed that pre-treatment with SHJKSmex significantly increased the Bcl-2/Bax ratio (*p* < 0.01, and *p* < 0.05, respectively) in the mouse model of ischaemic brain stroke in the 100 and 300 mg/kg groups (Figure 8(A)). The mice in the 300 mg/kg group showed significantly lower caspase-8 and −9 levels (*p* < 0.05, and *p* < 0.01, respectively) than the mice in the MCAO control group (Figure 8(B)). Thus, it was confirmed that SHJKSmex inhibits the intrinsic and extrinsic pathways of apoptosis through a mechanism that inhibits MCAO-induced brain injury.

### Effects of SHJKSmex 300 on histological changes during ischaemic brain tissue injury

**Figure 9.**
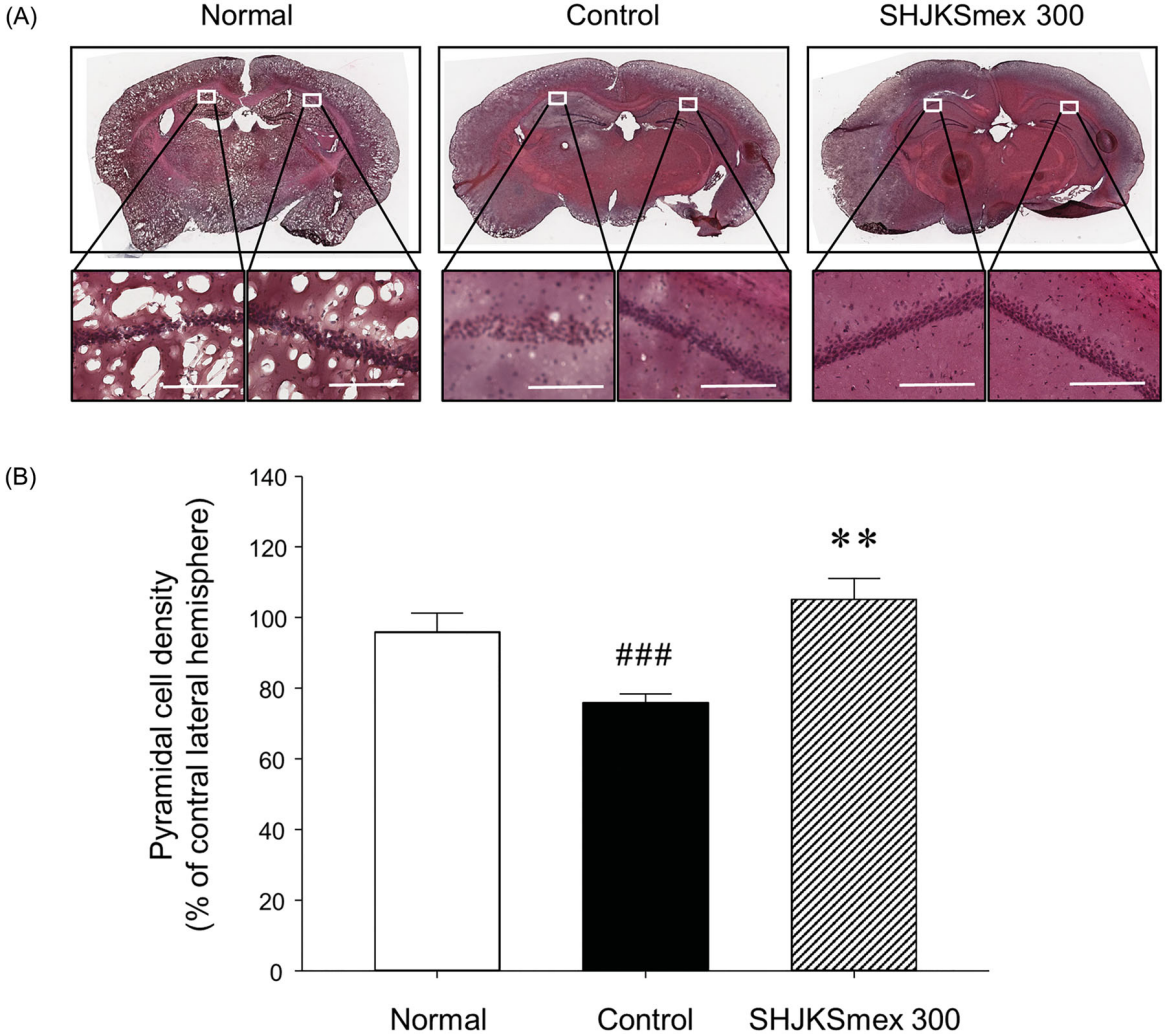
Representative photomicrographs of haematoxylin and eosin (H&E) staining (A) and quantitative analysis of the cell densities (B) of the hippocampal CA1 region in the brains of mice with middle cerebral artery occlusion (MCAO). Microscopic images of the whole brain (upper column) and the hippocampal CA1 region (lower column) from each group. Damaged cells were sparsely arranged. White bars in the lower column represent scale bars that indicate a length of 100 µm. Quantitative analysis showed significant changes in the pyramidal cell densities in the CA1 region. ^###^*p* < 0.001 vs. normal group and **p < 0.01 vs. control group; *n* = 5 in each group.

To confirm the histological changes in the brain tissue caused by MCAO and/or SHJKSmex treatment, we stained the tissue with H&E. The H&E staining results revealed that in the hippocampal CA1 area of the ischaemic mice, the cells were sparsely distributed relative to those in the sham-operated normal mouse brain (Figure 9). In the SHJKSmex 300 group, which was administered the highest SHJKSmex concentration in this study, the brain tissue was strongly stained with H&E and the cells in the hippocampal CA1 area were well aligned (Figure 9(A)). Thus, transient cerebral ischaemia led to an increase in cell death in the hippocampal CA1 region in the MCAO control group, and the rate of cell death was decreased in mice orally administered SHJKSmex 300 before MCAO. To evaluate the neuroprotective effects of SHJKSmex on hippocampal CA1 damage induced by MCAO, we measured pyramidal cell densities in the ipsilateral region compared to the contralateral pyramidal cell densities of the CA1 region. The pyramidal cell densities were significantly (*p* < 0.001) lower in the MCAO-induced group than in the sham-operated group; however, significantly (*p* < 0.01) higher densities were observed in the 300 mg/kg SHJKSmex group than in the MCAO control group (Figure 9(B)).

## Discussion

Ischaemic stroke is caused by the blockage of blood vessels in the brain and causes various speech and motor disorders or death (Donnan et al. 2008); thus, the prevention and treatment of stroke is crucial, as this condition reduces the quality of life of the patients and their families (Chamorro et al. 2016).

After ischaemic stroke, intravenous recombinant tissue plasminogen activator (r-tPA) should be administered to patients at the earliest time point within 4–5 h of symptom onset, and endovascular treatment may be considered for patients with internal carotid or MCAO within 6 h of symptom onset. Antiplatelet and anticoagulant therapies have been approved as secondary prevention strategies for ischaemic stroke. Non-vitamin K antagonist oral anticoagulation agents have been approved for stroke prevention in patients with non-valvular atrial fibrillation (Brien 2019).

In Korean medicine, cerebral ischaemia is included in the hit by wind (中風) phenomenon. Herbal medicine is used to enhance qi-blood (氣血) communication, dispel wind (祛風), regulate qi (調氣), and dispel phlegm (祛痰) (Kim et al. 1999). SHJKS, the first drug mentioned in the medical literature of Jejungsinpyeon (濟衆新編) in 1799, is a treatment agent that regulates qi and dispels phlegm (理氣去痰). SHJKS is also used to treat early stroke accompanied by symptoms of confusion, speech disorders, and motor disorders (Park and Lee 2007).

Employing the MCAO mouse model, Jeong and Lee (2008) revealed the neuroprotective effects of SHJKS, which was demonstrated by its regulation of Bax and Bcl-2 secretion. Kim and Kim (2006) also investigated the neuroprotective effect of SHJKS and revealed that it reduced Bax, caspase-3, HSP-72, and HIF-1α secretion. Moreover, Kim and Lee (Kim and Lee Kim and Lee 2001) found that SHJKS exhibited neuroprotective effects, decreased infarct volume and brain edoema, and increased the survival of pyramidal neurons in the CA1 area of the hippocampus.

Previous studies have reported that each medicinal herb has a different effect. In fact, Cyperi Rhizoma (香附子) derived from *Cyperus rotundus* Linné (Cyperaceae), Poria Sclerotium (茯苓) from *Poria cocos* Wolf (Polyporaceae), Pinelliae Tuber (半夏) from *Pinellia ternate* Breitenbach (Araceae), Citri Unshius Pericarpium (陳皮) from *Citrus unshiu* Markovich (Rutaceae), Atractylodis Rhizoma Alba (白朮) from *Atractylodes japonica* Koidzumi (Compositae), Angelicae Dahuricae Radix (白芷) from *Angelica dahurica* Bentham et Hooker f. (Umbelliferae), Arecae Pericarpium (大腹皮) from *Areca catechu* Linné (Palmae), Platycodonis Radix (桔梗) from *Platycodon grandifloras* A. De Candolle (Campanulaceae), Agastachis Herba (藿香) from *Agastache rugosa* (Fischer et Meyer) O. Kuntze (Labiatae), Magnoliae Cortex (厚朴) from *Magnolia officinalis* Rehder et Wilson (Magnoliaceae), Aucklandiae Radix (木香) from *Aucklandia lappa* Decne. (Compositae), and Arisaematis Rhizoma (天南星) from *Arisaema erubescens* Schott (Araceae) have been reported to exhibit anti-inflammatory and antioxidant effects (Han et al. 2011; Kim et al. 2013; Hong et al. 2014; Jung et al. 2014; Oh et al. 2014; Chang et al. 2015; Kang and Park 2015; Kim et al. 2015a; An et al. 2016; Kim et al. 2018; Kim and Park 2018; Kim et al. 2019).

SHJKS, the fundamental form of SHJKSm, is an herbal formula consisting of 15 medicinal herbs; however, this study used SHJKSm, which has been effectively used to treat ischaemic brain diseases such as ischaemic stroke and vascular dementia in Dong-Eui University Medical Centre, Busan, Republic of Korea. Five medicinal herbs were added to SHJKS to prepare the SHJKSm. The pharmacological activities of the newly added herbs are as follows: Siegesbeckia Herba (莃蘞) derived from *Siegesbeckia pubescens* Makino (Compositae) is reported to resolve blood stasis and exhibit an antioxidant effect (Kim et al. 2015b); Uncariae Ramulus et Uncus (釣鉤藤) derived from *Uncaria sinensis* Havil (Rubiaceae), Mori Radicis Cortex (桑白皮) from *Morus alba* Linné (Moraceae), and Bambusae Sulcus (竹瀝) from *Phyllostachys bambusoides* Sieb. et Zucc. (Gramineae) are reported to exhibit neuroprotective effects (Park et al. 2014; Chung et al. 2017; Geng et al. 2019); and Linderae Radix (烏藥) derived from *Lindera strychnifolia* Fernandez- Villar (Lauraceae) is reported to promote blood circulation (Lee et al. 2005).

When acute cerebral infarction occurs, the reduction in blood supply due to vascular occlusion results in the deprivation of oxygen and glucose, followed by an inflammatory response, ultimately causing blood–brain barrier (BBB) dysfunction and cell death (Liang et al. 2007; Donnan et al. 2008; Badaut et al. 2014; Chamorro et al. 2016). As a result of cerebral infarction, brain cells are oxidised; moreover, in severe cases, cell damage is irreversible, causing extensive apoptosis and necrosis. Accordingly, the treatment of acute cerebral infarction is based on suppressing the cascade damage caused by inflammation, oxidation, and apoptosis (Sander 2013; Wu et al. 2015).

In the present study, we evaluated the anti-inflammatory effects of SHJKSmex on ischaemia-induced brain damage in a mouse model. Based on our findings, pre-treatment with SHJKSmex effectively reduced the infarct volumes in the brains of mice in the SHJKS 100 and 300 groups (Figure 4A)). However, it did not significantly improve neurological behavioural deficits (Figure 4(B)). Neurobehavioral changes were assumed to be the same when brain damage was induced above a specific level, but the evidence could not be confirmed in this study. Thus, no significant results in neurological behavioural deficits could be observed because of the short duration of the study, and long-term and post-treatment experiments are warranted.

Haematoxylin can stain some tissue and cell structures, including the nucleus, whereas violet and eosin mainly result in pink staining of the cytoplasm (Ozawa and Sakaue 2020). In the present study, H&E staining results showed that cells were sparsely distributed in the hippocampal CA1 area of mice with MCAO compared to those in the sham-operated normal mouse brain. In the SHJKSmex 300 group, the brain tissue was strongly stained with H&E, the cells in the hippocampal CA1 area were well aligned (Figure 9(A)), and the density of pyramidal cells remained significantly high (Figure 9(B), demonstrating that the rate of cell death was decreased in the SHJKSmex 300 group. Based on histological staining, it was concluded that pre-treatment with SHJKSmex protected the nucleus and neuronal cells.

An increase in brain water content is caused by increased BBB permeability (Yao et al. 2015; Zhang and Warrington 2016) and correlates with AQP-4 expression (Iacovetta et al. 2012). In the present study, the mean brain edoema index was unchanged in the SHJKSmex groups (Figure 5(A)). However, the mean brain water content was significantly lower in the SHJKSmex 300 group (Figure 5(B)).

AQP-4 is one of the thirteen AQP subtypes derived from major endogenous proteins that facilitate the movement of water through the plasma membrane (Zelenina 2010). AQP-4 is important in the maintenance of water balance in the brain (Wang et al. 2014), and its high expression level is positively associated with the degree of edoema (Badaut et al. 2014). Thus, AQP-4 inhibition can effectively treat cytotoxic brain swelling in early cerebral infarction (He and Lu 2015). Although pre-treatment with SHJKSmex did not significantly suppress AQP-4 expression, the values showed a decreasing tendency (Figure 5(C)). Although there was no significant change in the mean brain edoema index, SHJKSmex could affect water channel-regulating factors, such as AQP-4, to decrease the brain water content and AQP-4 expression. However, further studies are necessary to confirm these results.

iNOS produces NO, which is a free radical that has different physiological and pathological effects. In the brain, an increase in iNOS after ischaemia leads to neuronal apoptosis by reducing the mitochondrial membrane potential of the neurons, promoting cytochrome C secretion, and activating caspase-9 and caspase-3 (del Zoppo et al. 2000; Nikoletopoulou et al. 2013). In the current study, pre-treatment with SHJKSmex caused a reduction in iNOS expression during MCAO-induced brain damage (Figure 7(A)).

After the initiation of ischaemia, energy depletion causes mitochondrial dysfunction and the release of ROS and reactive nitrogen species. ROS are also known to cause various diseases, such as ageing and cancer (Kehrer 1993; Ghanta et al. 2007), and their accumulation causes inflammation via the generation of harmful cell responses (Mukhopadhyay et al. 2012). MDA is a lipoperoxidation product whose levels are increased because of an increase in oxidative stress (Kotani et al. 2015). Lipid peroxide causes cell damage and ageing and alters the physiological and chemical properties of the cell membrane (Gutteridge 1995; Dawn-Linsley et al. 2005). In this study, pre-treatment with SHJKSmex effectively inhibited the increase in ROS and MDA levels caused by MCAO-induced brain injury. Furthermore, oxidative stress was significantly suppressed in the 300 mg/kg group (Figure 6). Our findings suggest that the effects of pre-treatment with SHJKSmex on ischaemic brain injury are associated with the inhibition of oxidative damage.

TNF-α exhibits neurotoxic effects by inducing apoptotic neuronal cell death and enhancing major histocompatibility complex class II and intercellular adhesion molecule 1 expression in astrocytes, leading to leukocyte infiltration and BBB breakdown. IL-1β directly induces the apoptosis of neuronal cells and enhances the expression of chemokines in microglia and astrocytes (Shichita et al. 2012). In the present study, pre-treatment with SHJKSmex effectively inhibited the increase in IL-1β caused by MCAO-induced brain injury (Figure 7(B)). Although not significantly, the expression of TNF-α was markedly decreased in the 300 mg/kg group (Figure 7(C)). Boutin et al. (2001) reported that cerebral infarct size decreased in the MCAO model established in IL-1B knock-out mice. SHJKSmex is presumed to inhibit the increase in IL-1β and thus reduce the cerebral infarct size.

Apoptosis is a programmed cell death mechanism in neurons that occurs during the development of the nervous system, chronic neurodegenerative diseases, and cerebral infarction caused by cerebral ischaemia. In apoptotic cell death, the Bcl-2 family and the cysteine proteases known as caspases serve as two important groups of enzymes (Gillardon et al. 1996; Wu and Tang 2016). Bcl-2 is an anti-apoptotic protein that regulates the apoptotic pathway and protects against cell death. Bax is a pro-apoptotic protein of the Bcl-2 family that is abundantly and selectively expressed during apoptosis and promotes cell death. The overexpression of Bcl-2 and downregulation of Bax represent anti-apoptotic processes (Gillardon et al. 1996; Wu and Tang 2016). Caspases are divided into initiator caspases (such as caspases-8 and −9) and executioner caspases (caspases-3, −6, and −7) according to their function and the sequence of their activation (McIlwain et al. 2015).

Intrinsic apoptosis is a mitochondrion-centered cell death that is mediated by mitochondrial outer membrane permeabilization, which causes apoptosome formation, activation of caspase-9, and the subsequent activation of effector caspases (Brentnall et al. 2013). In this study, pre-treatment with SHJKSmex significantly increased the Bcl-2/Bax ratio in the 100 and 300 mg/kg groups. Moreover, significantly lower levels of caspases-8 and −9 were found in the mice in the 300 mg/kg group than in those in the MCAO control group (Figure 8(B,C)). The increase in the Bcl-2/Bax ratio and decrease in caspase-8 and −9 levels demonstrated that pre-treatment with SHJKSmex resulted in anti-apoptotic effects in mice with ischaemic brain injury.

**Figure 10.**
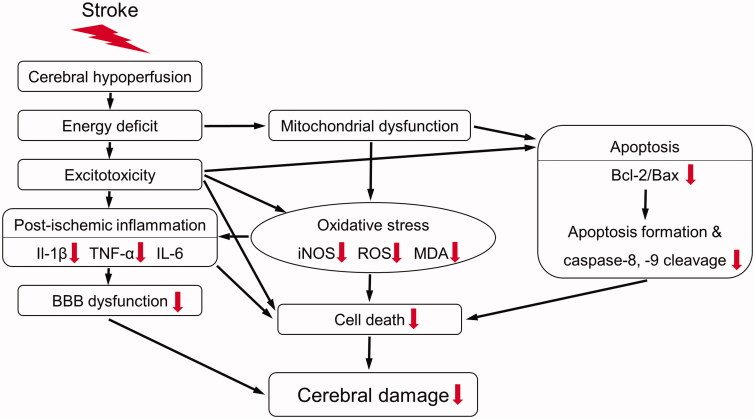
Schematic diagram of the antioxidant, anti-inflammatory, and anti-apoptotic mechanisms of the methanol fraction of the modified Seonghyangjeongki-san water extract (SHJKSmex) in mice with middle cerebral artery occlusion (MCAO). Red arrows represent the expected effects of SHJKSmex on the brain injury induced by MCAO.

Consequently, SHJKSmex pre-treatment restrained infarction in the mouse brain after MCAO by reducing the levels of oxidative stress induced by iNOS and ROS, thereby suppressing the expression of pro-inflammatory factors, such as IL-1β and TNF-α, and regulating the secretion of factors involved in apoptosis, such as caspases-8 and −9, Bcl-2, and Bax (Figure 10). Based on this finding, SHJKSmex may be regarded as a promising therapy for the prevention and initial treatment of stroke, as it can inhibit inflammatory responses in cerebral infarction.

The main mechanism of action for suppressing ischaemic stroke-induced brain damage in mice involves the process of reducing the inflammatory response by inhibiting the generation of ROS induced by ischaemia and reperfusion, and that of minimising apoptosis in the brain penumbra area by inhibiting the apoptosis process. In addition, with SHJKSmex administration, as the inflammatory response was suppressed, brain edoema was also reduced, resulting in less secondary damage. In the future, it will be necessary to study the protective effects of SHJKS on mitochondrial dysfunction, which is an upstream process of the mechanism confirmed in this study. Thus, further pharmacological studies are required to elucidate the additional mechanisms employed by SHJKSmex to inhibit brain damage, the effects of SHJKS on cerebral ischaemia-induced brain injury, and the mechanisms used by each SHJKS component to suppress the brain damage induced by ischaemia. Furthermore, experiments over a longer duration are necessary.

## Conclusions

To evaluate the effects of SHJKSmex pre-treatment on ischaemia-induced brain damage, infarct volumes of the brain, brain edoema indices, neurological deficit scores, and the expression of AQP-4, oxidative stress factors, inflammatory cytokines, and apoptotic proteins were measured 24 h after MCAO induction in mice. SHJKSmex pre-treatment significantly decreased the infarct volumes and brain water content and significantly inhibited the expression of ROS and MDA in the brains of mice after MCAO. The expression of iNOS and IL-1β was markedly suppressed by pre-treatment with SHJKSmex. The decreased protein expression ratio of Bcl-2/Bax was increased by SHJKSmex pre-treatment, and the expression levels of caspase-8 and caspase-9 were significantly inhibited in the brains of mice after MCAO induction. These findings indicate that SHJKS pre-treatment ameliorates ischaemic brain injury by suppressing apoptotic pathways. In conclusion, SHJKSmex pre-treatment reduced the infarct volumes in the brains of mice after MCAO by interfering with the inflammatory and apoptotic responses generated during ischaemic brain injury. Our findings indicate that SHJKS can prevent or treat cerebral ischaemia.
